# Supplementary material for: YOLOv8-Coal: a coal-rock image recognition method based on improved YOLOv8
Source: PeerJ Comput Sci. 2024 Sep 16;10:e2313. doi: 10.7717/peerj-cs.2313 (PMC11419675; doi:10.7717/peerj-cs.2313)
Supplement: Supplemental Information 1 [file peerj-cs-10-2313-s001.docx]

| **Stage 1: Traditional coal-rock image recognition methods** | | | | | |
| --- | --- | --- | --- | --- | --- |
| Reference | Model | Dataset | Data Processing Method | Advantages | Results |
| Sun & Su, 2013 | Fisher Discriminant Method | 80 images (40 anthracite images, 40 shale images) | Texture features are extracted using a gray level co-occurrence matrix (GLCM), extracting 22 texture features and reducing the dimensionality to 4. | Enhanced sample separability and reduced algorithmic time complexity. | The average recognition rate reached 94.12%. |
| Sun & Chen, 2015 | Asymmetric Generalized Gaussian Model Based on Wavelet Domain | 400 images (80 in the training set, 320 in the validation set) | Multi-level decomposition is performed using wavelet transform. | Effectively characterizes the texture features of coal rock images. | The average recognition rate reached 87.77%. |
| Wu & Tian, 2016 | Dictionary Learning Algorithm + KNN Algorithm | 208 images (156 in the training set, 52 in the validation set) | Dimensionality reduction is carried out using the PCA algorithm. | The extracted feature vectors exhibit improved sparsity. | The recognition rate for test samples reached 96.154%. |
| Wang & Zhang, 2020 | Local Binary Patterns (LBP) + Gray Level Co-occurrence Matrix (GLCM) | Images of coal and rock, specific numbers not mentioned | Local texture extraction is conducted using LBP, and a GLCM is implemented in four directions to extract four typical feature parameters. | Identifiable feature parameters suitable for coal rock classification are found, enhancing the robustness of coal rock identification. | Variance values of ASM and ENT are suitable for coal rock recognition. |
| Zhang et al., 2022 | Least Squares Support Vector Machine | 1800 images (900 coal images, 900 gangue images) | Grayscale images are processed using Gaussian filtering, extracting three features: grayscale skewness, grayscale variance, and texture contrast. | Strong feature extraction capabilities effectively address the issue of low identification rates in fully mechanized coal gangue mining. | The recognition accuracies for grayscale skewness and texture contrast were 92.2% and 91.5%, respectively. |
| **Stage 2: Convolutional neural network based coal rock image recognition methods** | | | | | |
| Reference | Model | Dataset | Data Processing Method | Advantages | Results |
| Huiling & Xin, 2019 | BP Neural Network | 180 images (bituminous coal, anthracite, sandstone, shale, each with 45 images) | Image features are extracted using wavelet transform. | Compared to traditional methods, it achieves a better recognition rate. | The recognition rate can reach 96.67%. |
| Liu et al., 2019 | Nearest Neighbor Classifier Using Chi-Square Distance | 4800 images (3840 in the training set, 960 in the validation set) | Multi-scale Completed Local Binary Patterns are extracted as texture information, followed by high-level macro information extraction using a CNN-based sub-model. The features are then fused. | Feature extraction using CNNs shows significant effects. | The accuracy reached 97.9167%. |
| Pu et al., 2019 | VGG16 Network | 240 images (200 in the training set, 40 in the validation set) | None | Transfer learning methods provide rapid and robust image differentiation without the need for extensive data support. | The accuracy reached 82.5%. |
| Si et al., 2020 | Improved CNN (Introducing Dropout, Weight Regularization, and Batch Normalization) | 6000 images, for 4-fold cross-validation | 300 original images (4032×3024) are segmented into 6000 images (256×256) with added noise, image scaling, and image rotation. | The improvements made solve the overfitting problem and accelerate the convergence speed. | The F1 score reached 78.62%. |
| **Stage 3: Coal rock image recognition methods based on two-stage detection approach** | | | | | |
| Reference | Model | Dataset | Data Processing Method | Advantages | Results |
| Hua, Xing & Zhao, 2019 | Faster R-CNN | 8000 images (5000 in the training set, 3000 in the validation set) | None | Achieved recognition and localization of coal rock images, moving beyond mere classification. | The mean Average Precision (mAP) approximately reached 88%. |
| Shan et al., 2022 | Improved Faster R-CNN: Introduced the Convolutional Block Attention Module (CBAM) for attention mechanisms. | 6000 images (3000 in the training set, 1500 in the validation set, 1500 in the test set) | Fuzzy set image enhancement preprocessing (dark channel dehazing, motion blur processing). | CBAM adjusts weight distribution during the backbone feature extraction process, significantly improving the network training accuracy. | The average precision and recall reached 82.63% and 86.53%, respectively, with an F1-score improvement of 7% over the original model. |
| Cao et al., 2024 | Improved Mask R-CNN: Proposed a Multichannel Forward-Linked Confusion Convolution Module, designed a multiscale high-resolution Feature Pyramid Network architecture, and proposed a multiscale Mask head structure. | 1500 images (1350 in the training set, 150 in the validation set) | None | The proposed improvements allow for serial propagation of feature information across different channels, merging with information flows at various scales, effectively enhancing segmentation precision and information processing efficiency. | The accuracy reached 97.38%, an improvement of 1.66% over the original model. |
| **Stage 4: Coal rock image recognition methods based on single-stage detection approach** | | | | | |
| Reference | Model | Dataset | Data Processing Method | Advantages | Results |
| Zhang et al., 2020 | YOLOv2 | 8000 images (5000 in the classification dataset, 3000 in the detection dataset) | Details unknown | Compared to two-stage detection methods, it achieves dual improvements in accuracy and speed. | The recognition accuracy reached 78%, with a detection speed of 63 frames per second. |
| Sun et al., 2022 | Improved YOLOv3: Introduced depthwise separable convolutions and utilized cubic spline interpolation for processing. | Details unknown | Median filtering, 90° rotation, brightness transformation, mirror flipping, and scaling. | Depthwise separable convolutions significantly reduce model parameter count, enhancing training and prediction speed. | The accuracy in the x and y directions improved by 5.85% and 16.99%, respectively, with a parameter reduction of about 80%, and prediction time decreased by about 5%. |
| Li et al., 2022 | Improved YOLOv3: Introduced deformable convolutions and optimized anchor box localization accuracy through averaging the results of multiple k-means clustering. | 2000 images (specific division unknown) | Random horizontal flipping, random cropping, random vertical flipping, and random changes to the brightness of the input image. | The improved model enhances anchor box localization accuracy and better captures local features of images. | The mAP reached 99.45%, with a maximum reduction in FLOPs of 61.4%. |
| Liu et al., 2021a | Improved YOLOv4: Optimized anchor values through clustering analysis on different datasets and increased the number of layers in the feature pyramid. | 20,283 images (8000 in the training set, 1125 in the validation set, 3450 in the test set) | Gaussian sum filtering algorithm and Laplacian operator. | The model captures richer detail information and exhibits good resistance to interference. | The mAP improved by 0.81% compared to before the improvements. |
| Wang et al., 2022 | Improved YOLOv5: Introduced the CBAM attention mechanism and Transformer. | Image count unknown (training set 60%, validation set 20%, test set 20%) | None | CBAM enhances feature expression in target areas and suppresses it in background areas. | The mAP can reach 92.8%. |
| Zhao & Deng, 2024 | Improved YOLOv7: Introduced ConvNext modules with 7×7 convolution kernels and SimAM attention mechanisms, employing αIoU as the loss function. | 3425 images (3243 in the training set, 172 in the validation set) | None | The introduction of attention mechanisms and modifications to convolutional modules enhance model performance. | The accuracy improved by 3.9%, the mAP by 1.5%, and the FLOPs decreased by 0.7G. |
